# Supplementary material for: Constructing, validating, and updating machine learning models to predict survival in children with Ebola Virus Disease
Source: PLoS Negl Trop Dis. 2022 Oct 12;16(10):e0010789. doi: 10.1371/journal.pntd.0010789 (PMC9555640; doi:10.1371/journal.pntd.0010789)
Supplement: S1 Text — (DOCX) [file pntd.0010789.s002.docx]

**S1 Text: Supplemental methods**

To test the assumption that data were missing completely at random (MCAR), Little’s test [1]

was performed separately on data from each EDP site. The null hypothesis that the data is

MCAR was rejected, prompting us to group the data by Ebola Treatment Centre (ETC) location and consider each separately. Of eleven ETC locations, Little’s test revealed data to be MCAR for four ETCs (Conakry, Bo Town, Lunsar, and Makeni), with the remaining five rejecting H0. A visual inspection of the missingness patterns for these locations revealed variables with high missingness at Monrovia ETC in Liberia and Nzérékoré ETC in Guinea, and variables that were not reported at all at Bongo, Margibi, and Kambia. Removal of high missingness or absent variables resulted in the data being MCAR, suggesting that non-randomness in the missing data can be attributed to geographical location and/or availability of data at each ETC.

We used the aregImpute function from the R package Hmisc [2] to create 100 imputed datasets.

In this approach, each imputation is generated by fitting a flexible additive model on a unique

bootstrap resample drawn from the original data. We fit a saturated imputation model using all

18 candidate predictors and allowed for nonlinear modeling of continuous variables using

restricted cubic splines.

Of all imputed variables, Ct value had the highest missingness at 47%. Supplementary Figure 1

shows the distribution of imputed Ct values, grouped by whether bleeding was reported within the first 48 hours. Patients who experienced bleeding had a higher viral load than those who did

not.

*Variable Selection and Model fitting*

The intermediate models generated during variable selection procedure were fitted with the glmnet() function in the R package glmnet [3].

Continuous variables were modeled as nonlinear terms using restricted cubic splines with three

knots. The logistic regression model was fit separately on 100 imputed datasets and the

coefficients were averaged to produce a final model using the lrm [4] and fit.mult.impute [2]

functions in the R packages rms and Hmisc, respectively.

**References**

[1] Little RJA. (1988). A Test of Missing Completely at Random for Multivariate Data with Missing Values, Journal of the American Statistical Association, 83:404, 1198-1202, DOI: [10.1080/01621459.1988.10478722](https://doi.org/10.1080/01621459.1988.10478722)

[2] Harrell, FE. (2019). "Package ‘hmisc’." CRAN2018 2019, 235-6

[3] Friedman J, Hastie T, Tibshirani R. (2010). “Regularization Paths for Generalized Linear Models via Coordinate Descent.” Journal of Statistical Software, 33(1), 1–22. doi: 10.18637/jss.v033.i01, https://www.jstatsoft.org/v33/i01/

[4] Harrell FE. (2021). rms: Regression Modeling Strategies. R package version 6.2-0.

<https://CRAN.R-project.org/package=rms>
