# Supplementary figures and images for: Constructing, validating, and updating machine learning models to predict survival in children with Ebola Virus Disease
Source: PLoS Negl Trop Dis. 2022 Oct 12;16(10):e0010789. doi: 10.1371/journal.pntd.0010789 (PMC9555640; doi:10.1371/journal.pntd.0010789)

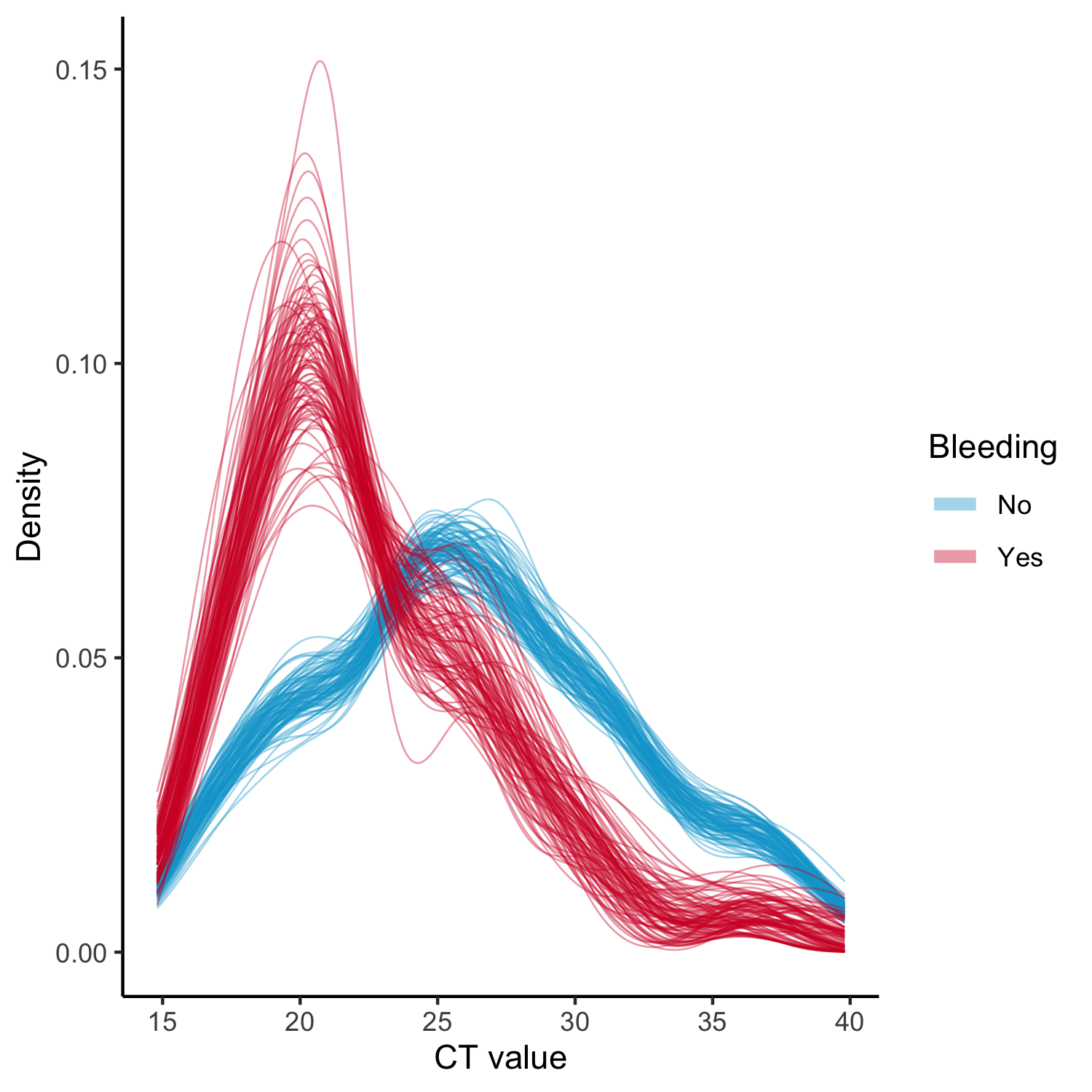

Supplement: S1 Fig — (TIF) [file pntd.0010789.s003.tif]

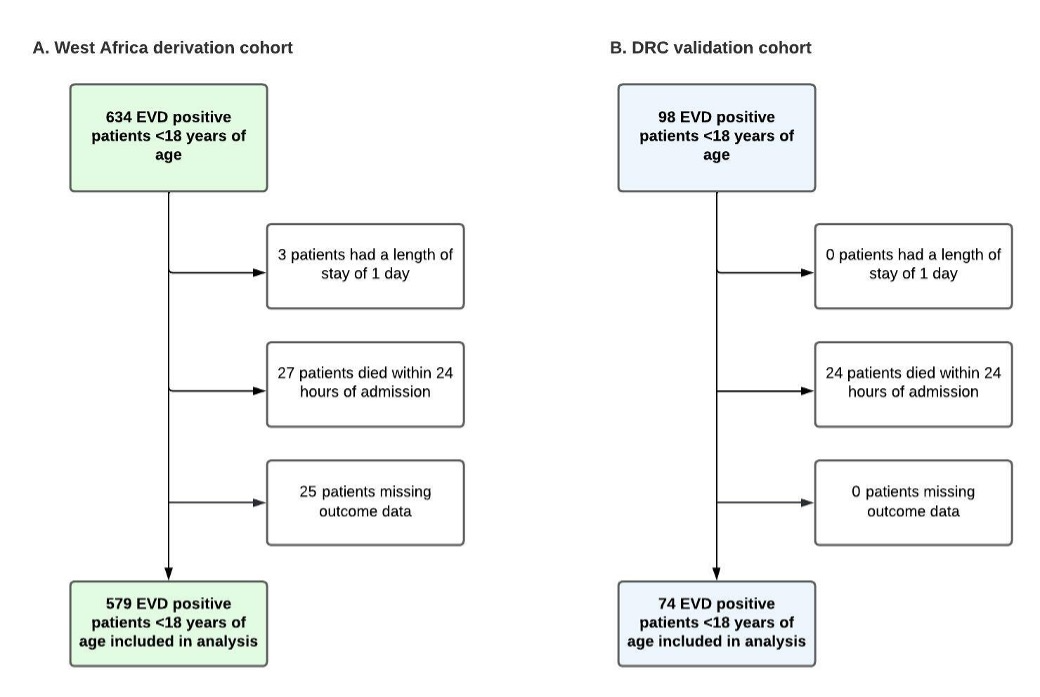

Supplement: S2 Fig — Flowchart of patients excluded in West Africa Derivation cohort (A) and DRC validation cohort (B). (TIF) [file pntd.0010789.s004.tif]

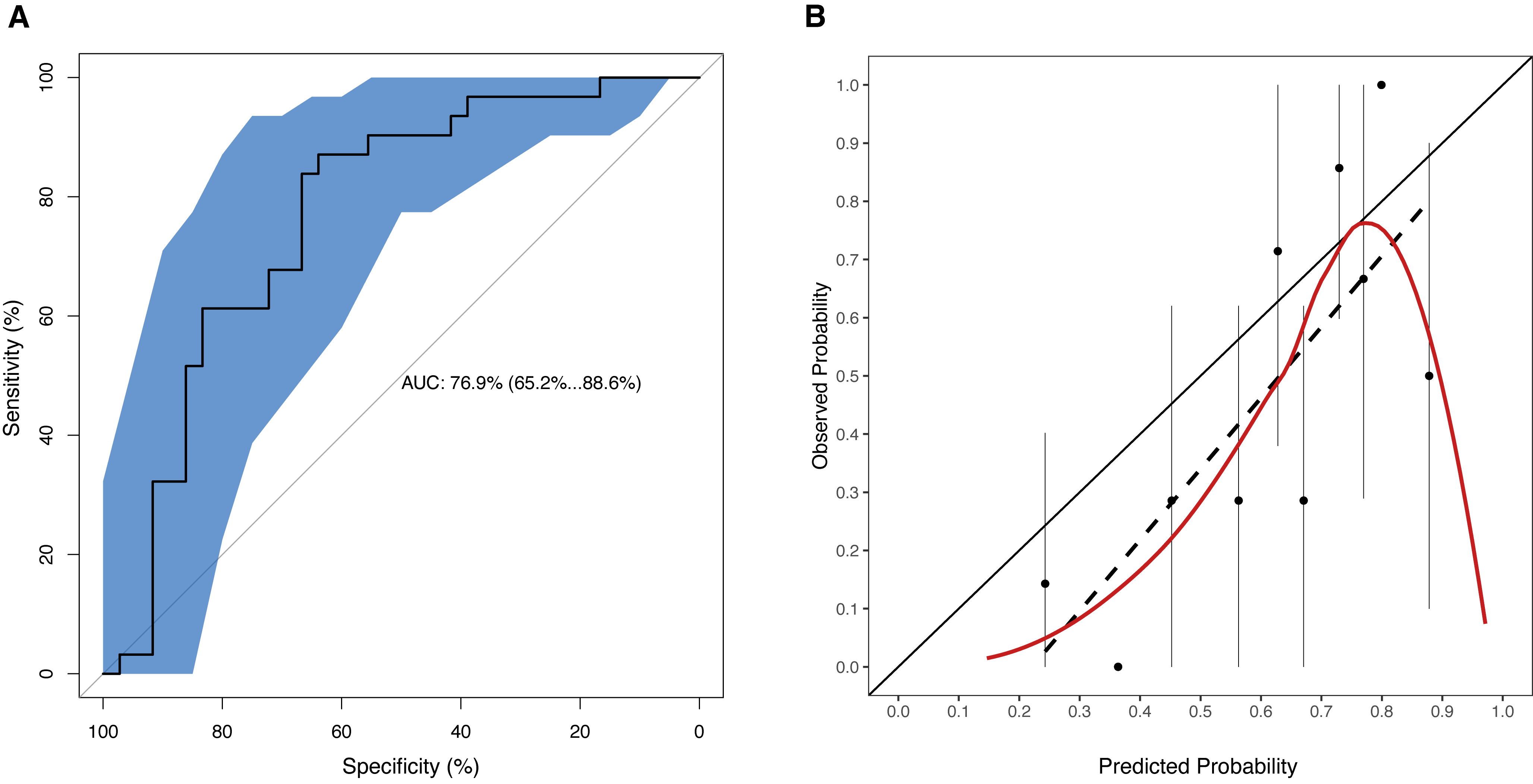

Supplement: S4 Fig — Receiver operating characteristic (ROC) (A) and calibration (B) plots for the minimal model (age+CT) described in [22], trained on all patients (not pediatric-specific) in the EVD West African dataset from IMC. The intercept and slope of the linear fit to the predicted probabilities are -0.27 and 1.22, respectively. (TIF) [file pntd.0010789.s006.tif]

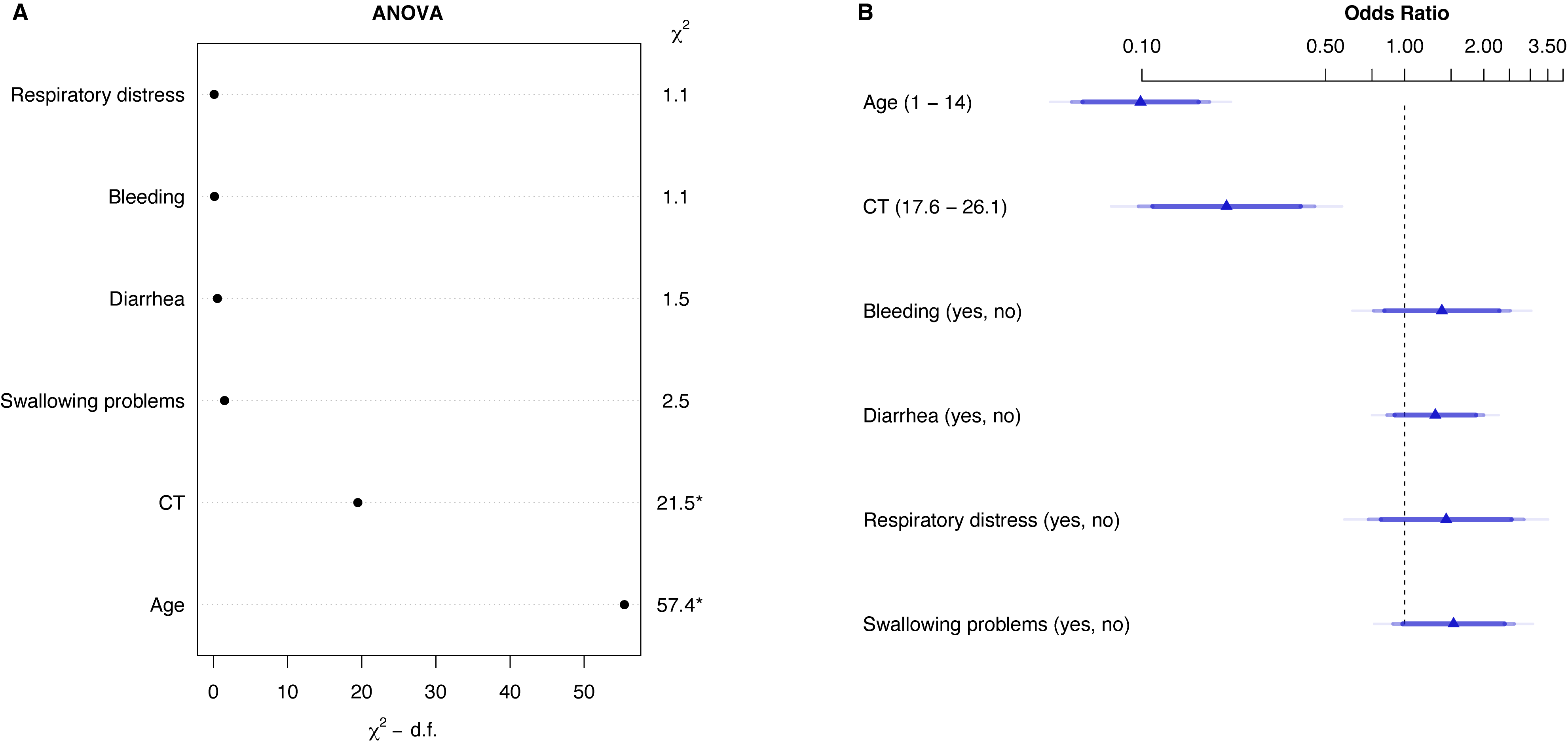

Supplement: S5 Fig — Analysis of variance chart generated with the anova() function in the rms package, showing a ranking of the features according to their predictive contribution to the model, as measured by the Wald χ2-d.f. (degrees of freedom) statistic (A). Chart generated with the summary function in rms, showing the odds ratios for all the features in the model, using interquartile-range odds ratios for continuous features (age and CT), and simple odds ratios for binary (yes/no) features (B). (TIF) [file pntd.0010789.s007.tif]
