# Supplementary material for: Constructing, validating, and updating machine learning models to predict survival in children with Ebola Virus Disease
Source: PLoS Negl Trop Dis. 2022 Oct 12;16(10):e0010789. doi: 10.1371/journal.pntd.0010789 (PMC9555640; doi:10.1371/journal.pntd.0010789)
Supplement: Attachment — Submitted filename: Pediatric.EVD.PLOS-NTD.Revised.ReviewerResponse-06-24-22.docx [file pntd.0010789.s013.docx]

Dear Editor,

We very much appreciate the comments of the Reviewers and believe that their suggestions have helped us to improve our manuscript. We provide responses to each reviewer’s suggestions below, written in blue.

Kind regards,

The Authors

Key Review Criteria Required for Acceptance

As you describe the new analyses required for acceptance, please consider the following:

Methods

*Reviewer #1:*

- There are several limitations to the methods. Many of the data elements were missing- in fact 10% of data were missing. Additionally there was a variety in the manner in which the patients were treated and data were collected. However, given the circumstances it is difficult to envision how the data could have been collected differently during the epidemic. The authors should mention which of the 18 variables were missing and in what percentages in the Supplemental material.
  - Thank you for the feedback. We agree that data collection during health emergency situations like these epidemics is extremely challenging. Missing data under these circumstances is almost inevitable, however, we applied robust statistical techniques to account for these issues, including multiple imputation and a novel variable selection method that uses regularized regression (Elastic Net) to reduce overfitting. We have added a table of the 18 variables that were used for variable selection with their respective percentages of missing values in the supplemental material (S1 Table).
- It would help if the authors provided more details about the IDDO EDP. Were data collected retrospectively or prospectively.
  - As described in the methods section of the manuscript, the IDDO EDP dataset consisted of retrospectively collected data from children presenting to Ebola treatment units (ETUs) in West Africa. It is the first global data repository for clinical, epidemiological, and laboratory data from patients with EVD during the 2014 - 2015 West Africa outbreak (specifically Liberia, Guinea and Sierra Leone) provided by the following organizations: Alliance for International Medical Action (ALIMA), International Medical Corps (IMC), Institute of Tropical Medicine Antwerp (ITM), Médecins Sans Frontières (MSF), University of Oxford, and Save the Children International (SCI), who had no role in the conduct of this study.
- Suggest providing more details about how information about symptoms were collected.
  - As detailed in the EVD Triage and Diagnosis of the Methods section, all organizations adhered to World Health Organization (WHO) diagnostic criteria and relevant national guidelines. Clinical and epidemiological data were collected in a systematic fashion for each of the organizations listed above.
- Suggest specifying why a 5 year age gap was used? Did the authors discuss dividing age into a categorical variable since a 4 year old is very different from an infant? Perhaps they could do a sensitivity analysis using age groups that are categorical (infant, toddler, etc.). Would also suggest including more details about the number of patients in each categorical age group. The IQR is presented but it would be helpful to have more details
  - A 5 year age gap was used as it is the widely accepted WHO categorization of age standards. Furthermore, the NIH also uses this age classification (references provided below). Table 4 of the Ahmad reference gives the percentage of the population in each 5-year age group in the WHO World Standard population.
- Ahmad O, Boschi-Pinto C, Lopez AD, Murray CJL, Lozano R, Inoue M. *Age standardization of rates: a new WHO standard* Geneva: World Health Organization; 2001.
- NIH. “World (WHO 2000-2025) Standard Population Data.” *NIH National Cancer Institute: Surveillance, Epidemiology, and End Results (SEER) Program*, https://seer.cancer.gov/stdpopulations/world.who.html.
- Please cite the reference for lines 156 regarding the Shapiro Wilk test.
  - Thank you for the suggestion. We have added the reference for line 156.

*Reviewer #2:*

- While Ebola virus disease (EVD) is well known to cause a highly lethal disease in all age groups, it is especially lethal young, pediatric patients. In this report, Genisca and colleagues use a machine learning approach to develop a prognostic model called the EVD prognosis in Children (EPiC). The initial EPiC model was created using a training dataset created using information based upon pediatric patient in the 2014-16 West Africa EVD outbreak. This resulted in a model that used age, Ct value, bleeding, diarrhea, breathlessness and dysphagia. This initial model performed with an AUC of 0.77 in the training cohort and when evaluated using a smaller validation cohort from the Democratic Republic of Congo (DRC) showed a similar performance. Further evaluation of the model in the validation cohort found that it tended to overestimate the risk of death. Since this performance was not optimal, two additional laboratory parameters, AST and CK, were investigated to determine whether adding their values to the model would improve performance. They found the predictive AUC was 0.87 to 0.90. Overall, the claims of this paper are supported by the data presented. However, parameters that were identified were already well known to be linked to poor outcomes and the predictive model was not translatable into a clinically useful tool that would allow physicians to easily use it to cohort patients based on risks.
  - Thank you for your insightful feedback. In order to make the model more clinically useful our team has developed an online calculator so that physicians can easily use it to calculate risk scores. This calculator can be found at: https://kelseymbutler.shinyapps.io/epic-calculator/.
- Line 121-123: Patient selection excluded subjects if they were missing outcome data or if they died within 24 hours of admission to the Ebola treatment unit, “as a prediction tool would not be useful for a moribound patient”. A flow chart/decision tree for each cohort that shows how many patients were excluded for each reason should be provided as a supplemental figure. This will help assess for any potential biases in their training and validation cohorts. This reviewer respectfully disagrees with the assumption that death within 24 hours is always associated with the patient being moribound at the time of admission or that there might not be a reversible condition that could be addressed if appropriate risk factors were identified early. This is because interventions could be targeted by clinicians to address the risk factors.
  - Thank you for your thoughtful comment. We respectfully disagree with the reviewer. Based on the experience in the field of some of the co-authors of this paper, we believe that inclusion of children who died within the first 24 hours could bias the model because the definitive diagnosis may not be confirmed and Ct values as well as most other laboratory results would not be available within that short timeframe. Furthermore, it may not be practical or meaningful for clinicians to apply such a model when they are focused on resuscitating an unstable moribund patient under challenging circumstances. Also, we constructed the model taking into account that patients may respond well to resuscitative measures (e.g., rehydration, glucose administration, electrolyte supplementation) within the first 24 hours, in which case, the model would not be accurate. We concluded that inclusion of these cases may have detracted from the model because of the large number of missing data. Therefore, taken together, we believe that by excluding unstable moribound children, we present a more robust and clinically meaningful model that is likely to be more accurate and generalizable to other settings. Additionally, per the reviewer’s suggestion a flow chart has been provided as a supplemental figure detailing how many patients were excluded from each dataset.
- While, the timing of the ALT and CK sample values used were indicated, it was not clear what time point was used to make the determination for the parameters evaluated in the models. That is, was it at admission, if the symptom developed at any point or something else. Similarly, for Ct, was the value at admission, the peak value or something else?
  - The AST, ALT, and CK values that we used represent the peak value measured within the first 48 hours after admission (we took this time window to maximize the number of patients with available biomarker data, while this data being reasonably close to the moment admission). For Ct, we used the value at admission. We performed a correlation analysis between all available peak biomarker values from the first 48 hours after admission and patient outcome, which yielded ALT, CK, and AST as the only biomarkers significantly associated with outcome at p<0.01. We omitted ALT from further analysis because it is highly collinear with AST.
- Were there differences in clinical site PCR testing in terms of the viral genes targeted and specific test that determined the Ct value? If so, how were differences accounted for in the model?
  - Thank you for your question. For the DRC site, all PCR cycle threshold (Ct) values presented in this study are based on RT-PCR of the same Zaire *ebolavirus* nucleoprotein locus using standardized RNA extraction procedures. For the West Africa sites, unfortunately it is unclear what the laboratory procedures were at each clinical site as the data were provided by several humanitarian organizations. When we re-ran all the calculations using normalized Ct values, all results remain unchanged (same selected variables from training data, model remaining performant on validation data, and increased performance of biomarker-augmented models) with the exception of the AUC for the validation, which is lower (0.71, CI [0.59, 0.84]). Both of these points have been added as limitations of the study.
- In the West African outbreak, some patients were able to receive experimental therapies under compassionate use, expanded access or as part of clinical trials. It would be helpful to see the number of patients in each group that received an experimental therapy, especially monoclonal antibodies.
  - Unfortunately, we do not have the data on experimental therapies for the West Africa outbreak. We only have detailed information for DRC, which we have added to Supplemental Table 3 (S3 Table). Since the number of patients in each therapeutic group was small (e.g., <20 per group), it was not possible to measure the effect size for each intervention or include it in the analytical model in a clinically meaningful way. This has been added as a limitation in the study.
- The manuscript notes that the original model overestimated the risk of death in the DRC validation cohort, this could be due to a larger number of patients in the validation cohort receiving effective therapeutics as part of expanded access (EUA) protocols or as part of the PALM randomized clinical trial. Particularly, two of the therapies were shown to be highly effective at reducing mortality. A supplementary table should be provided that shows the complete set of clinical parameters shown in Table 1 for the DRC cohort and that shows the number of patients in the cohort that received an experimental therapy under EUA or the PALM randomized clinical trial.
  - Thank you for your feedback. A supplementary table of the clinical parameters you described above has been included (S3 Table).
- The results section discussing the Derivation of the Clinical Prognostic Model (lines 231-235) states what parameter were used in the model but does not explain to the reader how those were chosen amongst all the significantly different variables shown in Table 1. The manuscript would be improved by adding a few more details on how the machine learning protocol reduced parameter sets to two continuous and 4 binary covariates.
  - Thank you for the feedback. The section the Reviewer is referring to is in the Results section of the manuscript. Details on how the final variables were selected for the model is detailed in the Methods section under the “Variable selection” subsection. In short, the binary variable selection protocol is as follows: Elastic Net was applied to each imputed dataset, the sign of the coefficients of the binary symptom variables in the resulting models were tallied, and those variables with the percentage of positive model coefficients above a given threshold were selected (Supplementary Table 2). This selection criterion facilitated the inclusion of groups of correlated predictors and predictors with small but significant effects. The threshold for variable inclusion was set at 100% to exclude variables with weak and/or inconsistent effects. Based on this protocol, any bleeding, dysphagia, breathlessness, and diarrhea were included in the EPiC model. Age and Ct were selected for inclusion in the model as they are highly correlated with poor prognosis in children with (references cited below).
    - Smit MA, Michelow IC, Glavis-Bloom J, Wolfman V, Levine AC. Characteristics and Outcomes of Pediatric Patients With Ebola Virus Disease Admitted to Treatment Units in Liberia and Sierra Leone: A Retrospective Cohort Study. Clin Infect Dis. 2017 Feb 1;64(3):243-249. doi: 10.1093/cid/ciw725. Epub 2016 Oct 25. PMID: 28011610; PMCID: PMC5241778.
    - Shah T, Greig J, van der Plas LM, Achar J, Caleo G, Squire JS, Turay AS, Joshy G, D'Este C, Banks E, Vogt F, Lokuge K. Inpatient signs and symptoms and factors associated with death in children aged 5 years and younger admitted to two Ebola management centres in Sierra Leone, 2014: a retrospective cohort study. Lancet Glob Health. 2016 Jul;4(7):e495-501. doi: 10.1016/S2214-109X(16)30097-3. PMID: 27340004.
    - Chérif MS, Koonrungsesomboon N, Kassé D, et al. Ebola virus disease in children during the 2014-2015 epidemic in Guinea: a nationwide cohort study. Eur J Pediatr. 2017 Jun;176(6):791-796. doi: 10.1007/s00431-017-2914-z. Epub 2017 Apr 25. PMID: 28444452.
    - Gulland A. Ebola mortality is highest among babies, finds study. *BMJ*. 2015;350(mar27 10):h1718-h1718. doi:10.1136/bmj.h1718.
- Line 252-253 indicated that ALT was not used in the models and that ALT or CK were. This was because ALT was highly correlated with AST. Therefore, all comments and statements in the manuscript that ALT was used should be removed. For example on lines 280.
  - Thank you for the suggestion. All comments and statements related to using ALT have been removed in the manuscript.
- Line 312-313. It is not clear from this report how clinician would use this model to predict risk because there is not a toolkit online or clinical scoring system or something similar that one would utilize to calculate a risk. Also, the formal definitions of how the clinical parameters used were defined is not available. For example, what is the formal definition of breathlessness? Is it a subjectively determined or is it an abnormal respiratory rate or is it having signs of severe respiratory distress or something else. Thus, statement about ease of application of the mode should be removed.
  - Thank you for the feedback. Based on your suggestion, we developed an online calculator so that physicians can easily use it to calculate risk scores. This calculator can be found at <https://kelseymbutler.shinyapps.io/epic-calculator/> and a link has been included in the text: As stated in the methods section of the manuscript, clinicians at each Ebola treatment center followed the World Health Organization’s guidelines for clinical assessment and definitions of abnormal signs and symptoms e.g. The WHO manual refers to age-related respiratory rates for respiratory distress. We cited the WHO guidelines in our Reference list (#19 and #42).
- Line 239. Validation AUC is 0.79 on this line and in the abstract is it 0.76
  - Thank you for bringing this to our attention. The validation AUC is 0.76, as stated in the abstract and presented in Figure 2. The AUC has been corrected in the manuscript.
- Figure 2B. The figure would be improved by providing a description in the legend or by labels in the panel for what the dashed and red lines are.
  - Thanks for pointing this out. We have added a detailed legend explaining the elements presented in each plot, as well as references in the main text for the R packages and method used to generate them.

*Reviewer #3:*

- It is not clear why there is a need for a new model. As also the authors state in the manuscript, there are existing predictive models. These models were not evaluated using training and validation sets separately. The same datasets could be used to train, test and validate the existing predictive models. At least to justify the need for a new predictive model the proposed model could be compared to these existing models over the same dataset.
  - Thank you for the feedback. As explained in the introduction section, the previously developed predictive models are limited in geographic and temporal scope. Furthermore, to our knowledge there are no published pediatric-specific EVD prediction models. Our intention for developing the EPiC model was to create a pediatric specific predictive model, making it the first predictive model for children under 18 years of age. Additionally, the EPiC model is also the first prognostic model to be externally validated, as none of the previously developed models for adults have been externally validated. As such, the EPiC model is more generalizable to other pediatric populations.
- Bootstrapping is used for model training (or derivation t as authors denote in the manuscript). This approach could overestimate the training performance. Why not use K-fold cross validation?
  - Thanks for suggesting the use of K-fold cross validation. We did not include it because our bootstrap performance numbers are already optimism-corrected. We calculated bootstrap estimates that consider the bias due to overfitting or the “optimism” in the final model fit as discussed in Efron and Gong’s article “A leisurely look at the bootstrap, the jackknife, and cross-validation”.
    - Ref: Efron B, Gong G. A Leisurely Look at the Bootstrap, the Jackknife, and Cross-Validation. 1983. *The American Statistician*, *37*(1), 36–48. https://doi.org/10.2307/2685844
- It will be good to prepare a table that includes all the variables that are used in Elastic net for variable selection. Also this table could include information about if these variables are continuous valued or binary.
  - Thank you for the suggestion. An additional supplemental table has been included to reflect the above suggestions (S2 Table).

Results

*Reviewer #1:* The data presented do match the analysis plan and the figures are legible and clear. A few minor suggestions:

- In Table 1, I would add a footnote mentioning that the OR is for each 5 year increase in age.
  - Thank you for the suggestion. Table 1 has been updated to reflect this.
- Suggest a new title for Table 1 reflecting that this title is focused on mortality and predictors of mortality.
  - Thank you for your feedback. The table in question presents data not just for cases who died but also those that survived as such the authors believe the current title best reflects the data it presents.
- The mortality, location, and age of the validation cohort appear to be very different from the original cohort. Do the authors think this might affect their results?
  - Thank you for your question. It is important to validate a model in different cohorts with diverse characteristics and from distinct geographic locations in order to test its accuracy and performance on an independent dataset. In fact several studies (cited below) have stressed that models should not be recommended for clinical use prior to validation, as models may be overfit to the population from which they were derived. Therefore, we consider validation of our model in a dissimilar cohort to be a strength of this paper.
    - Steyerberg EW. Clinical Prediction Models: A Practical Approach to Development, Validation, and Updating. Springer; New York, 2009.
    - Debray TP, Vergouwe Y, Koffijberg H, Nieboer D, Steyerberg EW, Moons KG. A new framework to enhance the interpretation of external validation studies of clinical prediction models. J Clin Epidemiol. 2015 Mar;68(3):279-89. doi: 10.1016/j.jclinepi.2014.06.018. Epub 2014 Aug 30. PMID: 25179855.
    - Moons KGM, Kengne AP, Grobbee DE, Royston P, Vergouwe Y, Altman DG, et al. Risk prediction models: II. External validation, model updating, and impact assessment. Heart 2012;98(9):691e8.
    - Altman DG, Vergouwe Y, Royston P, Moons KGM. Prognosis and prognostic research: validating a prognostic model. BMJ 2009; 338:b605.
    - Reilly BM, Evans AT. Translating clinical research into clinical practice: impact of using prediction rules to make decisions. Ann Intern Med 2006;144:201209

*Reviewer #2*: see above

*Reviewer #3*:

- It is not clear how many patients were in the training and how many were in the validation? Tables 1 and 2 are confusing, as the number participants used for derivation in Table 2 is 234 while total number of patients in external validation set is 74.
  - Thank you for your feedback. Table 1 presents data on patients who survived and died for the West Africa derivation cohort (total 579 patients, including 234 that died). Table 2 compares baseline characteristics of those patients who died in both the West Africa derivation cohort (234) and DRC validation cohort (74). We’ve included a flow chart in the supplementary material for further clarification.
- What does derivation cohort presented in Table 2 mean? Is the derivation cohort from DRC Mangina dataset used to refine the model trained using the data described in Table 1? If yes, the results do not present an external validation. Evaluation data should not be used in the training.
  - Thank you for your questions. The derivation cohort is from the West Africa dataset and the validation cohort is from the DRC dataset as explained in the text under the methods section of page 5. Table 1 presents demographic and clinical characteristics of patients included in the West Africa derivation cohort only. Table 2 was used to compare the differences in baseline characteristics between the two cohorts.
- Overall description of results need to be more clear, more clear description of Tables are needed.
  - Thank you for the suggestion. We have added a clearer description of the tables in the title and manuscript.
- Why not train and test the existing predictive methods using the same datasets to compare with the proposed method? This will also justify the need for a new predictive model.
  - Thank you for the feedback. This is a similar comment to the one that Reviewer 3 proposed above. As stated previously and in the manuscript, we decided not to test existing predictive models as those predictive models are limited in geographic and temporal scope. Furthermore, to our knowledge there are no published pediatric-specific EVD prediction models. Our intention for developing the EPiC model was to create a pediatric specific predictive model, making it the first predictive model for children under 18 years of age.
- How are the Sensitivity vs Specificity curves obtained? This needs be explained clearly.
  - We used the function plot.roc() from the package pROC to generate the AUC in R, which measures sensitivity vs specificity curves of the binary predictor for a sliding prediction threshold between 0 and 1.
    - Reference: Robin X, Turck N, Hainard A, Tiberti N, Lisacek F, Sanchez J, Müller M (2011). “pROC: an open-source package for R and S+ to analyze and compare ROC curves.” BMC Bioinformatics, 12, 77.
- Also, what are the probability of false alarm and probability of miss?
  - Thank you for your question. The confusion matrix for predictions at the optimal accuracy cutoff (0.63) and a table detailing additional performance measures, including false alarm and probability of miss, were incorporated as a pair of supplementary tables (S4a & S4b).

Conclusions

*Reviewer #1*:

- The authors did not include treatment type or type of facility in their analysis. Do the authors think this could be a potential limitation of their study?
  - All patients were treated at Ebola treatment centers both in the DRC and West Africa. While there were treatment differences in the transit or holding centers that manage the suspected cases, all confirmed cases were moved to treatment centers, which is where our data came from. All West Africa’s treatment protocols were the same at these centers; as stated on page 6 “All organizations adhered to World Health Organization (WHO) diagnostic criteria and relevant national guidelines”. However, there may have been slight differences in treatment protocols in West Africa’s treatment centers versus DRC’s treatment centers but not in the type of facilities. This has been added to the limitations section.
- Why do the authors think that asthenia, headache, and abdominal pain were correlated with better outcomes? Could this have to do with the ability of the child to relate their symptoms to a caregiver? In other words a child with a better mental status or who is older may be better able to express those symptoms?
  - Thank you for your insightful questions. We took a closer look into the interactions between several variables (abdominal pain, asthenia, headache, muscle and bone pain) and age. We found these variables to be correlated with age, illustrating that children in the pre-verbal age group (defined as <2 years) cannot reliably report this symptom. The results from this analysis have been added to the supplemental material and this has been added as a limitation.
    - Ref: Bright Horizons. “Look Who’s Talking: Speech Development for Children”. Accessed: 20 June 2022. https://www.brighthorizons.com/family-resources/articles/e-news/look-whos-talking-childrens-development-of-speech
- Authors should mention that collecting symptom information in children is difficult and may be a limitation (especially for very young children)
  - Thank you for your feedback. This limitation was added to the limitation section.
- Line 283- the authors should mention specifically that shock may lead to an increase in AST/ALT and CK
  - A sentence has been added to the discussion section to address the above suggestion.
- Another limitation that the authors should mention is that Ct values vary from assay to assay
  - Thank you for your suggestion. This limitation has been added to the paragraph.
- The validation model only looked at 74 cases in a different setting and time frame which is an additional limitation
  - We have clarified that the validation dataset included only 74 cases.

*Reviewer #2*: see above

*Reviewer #3*: (No Response)

Editorial and Data Presentation Modifications

*Reviewer #1:* (No Response)

*Reviewer #2:* (No Response)

*Reviewer #3:* (No Response)

Summary and General Comments

*Reviewer #1*: This is a well written manuscript that provides a model for assessing risk of death in pediatric patients with EVD. This is an important study because it can prove helpful in future epidemics. It would be further strengthened if it included data on therapeutics and care.

*Reviewer #2:* (No Response)

*Reviewer #3:* (No Response)
