# Supplementary material for: Constructing, validating, and updating machine learning models to predict survival in children with Ebola Virus Disease
Source: PLoS Negl Trop Dis. 2022 Oct 12;16(10):e0010789. doi: 10.1371/journal.pntd.0010789 (PMC9555640; doi:10.1371/journal.pntd.0010789)
Supplement: Attachment — Submitted filename: Pediatric.EVD-PLOS-NTD.Revision.Response-08-21-22.docx [file pntd.0010789.s014.docx]

We appreciate the last comments of the Reviewers and believe that their suggestions have helped to further improve our manuscript. Responses to reviewer’s suggestions are written in blue.

Key Review Criteria Required for Acceptance

As you describe the new analyses required for acceptance, please consider the following:

Methods

*Reviewer #2:* see editorial and date presentation section

*Reviewer #3:*

- The authors addressed most of our concerns. However, we still think that a comparison with the existing methods will motivate the need for a new predictive model better. The authors claim that the previously developed predictive models are limited in geographic and temporal scope. Our understanding is that the previous models while being trained and tested did not have access to the datasets that the authors used in this manuscript. When trained and tested in the same dataset, these existing models may outperform the methodology that the authors are proposing in this paper. We think that the authors need to demonstrate that the predictive model they are proposing is outperforming the existing methods when the same datasets are used. This will provide a better motivation for the proposed methodology. Then if the proposed method outperforms the existing methods when used on the same dataset, a discussion on methodological differences and why these differences matter should be discussed.
  - We appreciate the reviewer’s thoughtful feedback. Though we understand the benefits of comparing the performance of each model, the EpiC model is the first pediatric specific prognostic model. The models that exist (Hartley MA, et al 2017; Colubri A, et al 2019; Kangbai JB, et al 2020) are for those across all ages so this comparison is not straightforward, and often times simply not possible because models may use different features. We have highlighted this point on page 5 of our manuscript to make it clear to readers. However, we were able to apply the minimal model (age+CT) from Colubri A, et al 2019, and we observe that our new model exhibits better calibration (page 14 and 15), although AUC is similar (new Figure S4). The similar AUC is not surprising, since it stems from the fact that clinical features have a much smaller importance in the model relative to age and viral load, a fact that has been observed in prior studies as well including Colubri , at al. However, our additional odd ratios analysis (Figure S6) shows that, even when their contributions are small, the clinical features identified by our variable selection protocol all have a positive OR, which also points to the ability of our protocol to find meaningful features for inclusion in the model, even when they are weakly associated with the dependent variable. Finally, it is worth noting that when the EPiC model is augmented with both the AST and CK biomarkers, discriminative performance is nearly perfect (page 17). Of course, these results should be taken as preliminary since the number of patients with all the necessary lab data is very small, and validation will be required in follow-up studies.

Results

*Reviewer #2:* see editorial and date presentation section

*Reviewer #3:* please see above.

Conclusions

*Reviewer #2:* see editorial and date presentation section

*Reviewer #3:* please see above.

Editorial and Data Presentation Modifications

*Reviewer #2:* If not commented on below, the responses provided by the authors has satisfied this reviewer.

- Initial Reviewer comment: Line 121-123: Patient selection excluded subjects if they were missing outcome data or if they died within 24 hours of admission to the Ebola treatment unit, “as a prediction tool would not be useful for a moribound patient”. A flow chart/decision tree for each cohort that shows how many patients were excluded for each reason should be provided as a supplemental figure. This will help assess for any potential biases in their training and validation cohorts. This reviewer respectfully disagrees with the assumption that death within 24 hours is always associated with the patient being moribound at the time of admission or that there might not be a reversible condition that could be addressed if appropriate risk factors were identified early. This is because interventions could be targeted by clinicians to address the risk factors.
  - Author Response: Thank you for your thoughtful comment. We respectfully disagree with the reviewer. Based on the experience in the field of some of the co-authors of this paper, we believe that inclusion of children who died within the first 24 hours could bias the model because the definitive diagnosis may not be confirmed and Ct values as well as most other laboratory results would not be available within that short timeframe. Furthermore, it may not be practical or meaningful for clinicians to apply such a model when they are focused on resuscitating an unstable moribund patient under challenging circumstances. Also, we constructed the model taking into account that patients may respond well to resuscitative measures (e.g., rehydration, glucose administration, electrolyte supplementation) within the first 24 hours, in which case, the model would not be accurate. We concluded that inclusion of these cases may have detracted from the model because of the large number of missing data. Therefore, taken together, we believe that by excluding unstable moribound children, we present a more robust and clinically meaningful model that is likely to be more accurate and generalizable to other settings. Additionally, per the reviewer’s suggestion a flow chart has been provided as a supplemental figure detailing how many patients were excluded from each dataset.
  - Reviewer response: The way the section on participant selection is written that is being discussed, it is appears to assume that someone who dies in the first 24 hours is moribund at the time of admission. Could they not become moribund after admission but within the first 24 hours? In that case, having a tool available to predict which ones might become moribund within the first 24 hours might be useful. That said, I would be satisfied if the authors would rewrite this sentence to be more consistent with the authors response above.
  - Thank you for your feedback. We have revised our methods section on page 6 as suggested by the reviewer to simply state that patients who died within one day of admission were excluded. While having a tool available to predict which patients might become moribund within the first 24 hours would be useful, unfortunately our data from which we derived the model from does not have time of death, so it is not possible to determine if the patient died immediately upon arrival or many hours later on the day of admission. We recognize that this may be a limitation of our study and have added it to the limitation section to reflect this.
- Thank you for providing the data on excluded patients in each cohort. The DRC validation cohort has ~24% of patients excluded, whereas as the West Africa had ~4%. This could influence the outcomes being analyzed. For example, could the overestimation of death in the DRC cohort be because of the higher proportion of patient who died in the first 24 hours being excluded? Some comment should be provided in the manuscript.
  - Thank you for raising this important question. It is true that a proportionately higher number of patients were excluded from the DRC dataset (24/98 = 24%) compared to the West Africa dataset (55/634=9%). The EPiC model exhibits good calibration overall, with only a small bias towards overestimating risk across all patients (by around 10%). It’s unlikely that excluding a higher risk group of children (those who died within one day) would have affected the calibration in this manner, as we would then expect a worse calibrated model with underestimation only for higher risk patients. As mentioned above, we have added the high rate of exclusion due to death in the first day to our limitations section and a brief remark about the model calibration in the discussion (page 20).
- Initial Reviewer comment: The results section discussing the Derivation of the Clinical Prognostic Model (lines 231-235) states what parameter were used in the model but does not explain to the reader how those were chosen amongst all the significantly different variables shown in Table 1. The manuscript would be improved by adding a few more details on how the machine learning protocol reduced parameter sets to two continuous and 4 binary covariates.
  - Author response: Thank you for the feedback. The section the Reviewer is referring to is in the Results section of the manuscript. Details on how the final variables were selected for the model is detailed in the Methods section under the “Variable selection” subsection. In short, the binary variable selection protocol is as follows: Elastic Net was applied to each imputed dataset, the sign of the coefficients of the binary symptom variables in the resulting models were tallied, and those variables with the percentage of positive model coefficients above a given threshold were selected (Supplementary Table 2). This selection criterion facilitated the inclusion of groups of correlated predictors and predictors with small but significant effects. The threshold for variable inclusion was set at 100% to exclude variables with weak and/or inconsistent effects. Based on this protocol, any bleeding, dysphagia, breathlessness, and diarrhea were included in the EPiC model. Age and Ct were selected for inclusion in the model as they are highly correlated with poor prognosis in children with (references cited below)..[...]
  - Reviewer response: Thanks for your response. I appreciate the details are in the methods. The comment was to help the reader of the manuscript better follow the logic without needing to refer back to the methods section. The recommendation stands but it is not a requirement.
- Initial Reviewer comment: Line 312-313. It is not clear from this report how clinician would use this model to predict risk because there is not a toolkit online or clinical scoring system or something similar that one would utilize to calculate a risk. Also, the formal definitions of how the clinical parameters used were defined is not available. For example, what is the formal definition of breathlessness? Is it a subjectively determined or is it an abnormal respiratory rate or is it having signs of severe respiratory distress or something else. Thus, statement about ease of application of the mode should be removed.
  - Author Response: Thank you for the feedback. Based on your suggestion, we developed an online calculator so that physicians can easily use it to calculate risk scores. This calculator can be found at [https://kelseymbutler.shinyapps.io/epic-calculator/ [kelseymbutler.shinyapps.io]](https://urldefense.com/v3/__https:/kelseymbutler.shinyapps.io/epic-calculator/__;!!Fv-PpA!R-dSHw4Y5T0NSTBPRtkv5kNmHhkJ-91wWGTE788Ihv154nEeuI2M4BYN-3uscwzun04d3gS2XtHVHhnpVruz9hf5nssKpA$) and a link has been included in the text: As stated in the methods section of the manuscript, clinicians at each Ebola treatment center followed the World Health Organization’s guidelines for clinical assessment and definitions of abnormal signs and symptoms e.g. The WHO manual refers to age-related respiratory rates for respiratory distress. We cited the WHO guidelines in our Reference list (#19 and #42).
  - Reviewer Response: Thank you for providing the online calculator. The link for reference 19 needs to be updated. While the presence of bleeding, diarrhea and dysphagia can reasonably be pulled from a chart breathlessness is more subjective. In addition, the WHO documents discussed do not contains a definition for breathlessness. Respiratory distress is defined. However, even if breathlessness was defined in the WHO documents, a reader should not have to go to the WHO guidelines to determine what was meant. Please provide the formal definitions used in your models in the manuscript.
  - Thank you for your feedback the link to reference 19 has been updated. With regards to the definition for breathlessness, this term was taken from the West Africa dataset, from which the model was derived. As mentioned in the text, this dataset was collated from several different humanitarian agencies with differing data collection and laboratory procedures. This is why there is a discrepancy in the variable name between the data and WHO documents. Furthermore, the 2016 WHO guidance, equated the terms “difficulty breathing” with “respiratory distress”. Nonetheless, we’ve changed all “breathlessness” terms to “respiratory distress” as to avoid any further confusion and defined in the text.

*Reviewer #3:* (No Response)

Summary and General Comments

*Reviewer #2:* see editorial and date presentation section

*Reviewer #3:* (No Response)
